# Analysis of influencing factors and predictive performance of lumbar disc herniation in elderly patients with osteoporosis

**DOI:** 10.3389/fendo.2026.1885640

**Published:** 2026-07-31

**Authors:** Zheng Gong, Kun Shi, Weiwei Meng, Yinjuan Tang, Zhipeng Song, Shaohua Chen, Shunchao Liu, Jiyang Zhu, Jun Zhuang, Wencheng Ye, Xiaonan Liu

**Affiliations:** Department of Orthopedics and Traumatology, Tianshan Hospital of Traditional Chinese Medicine, Shanghai, China

**Keywords:** elderly, lumbar disc herniation, osteoporosis, predictive performance, risk factors

## Abstract

**Objective:**

This study aimed to analyze the influencing factors associated with lumbar disc herniation (LDH) in elderly patients with osteoporosis and to evaluate the predictive performance of multiple clinical indicators for LDH risk, thereby providing evidence for early risk assessment and targeted prevention and treatment strategies.

**Methods:**

A single-center retrospective case–control study was conducted at Tianshan Hospital of Traditional Chinese Medicine, Changning District, Shanghai, China. Clinical data of patients aged 60–80 years who underwent lumbar quantitative computed tomography (QCT) examination from January 2023 to January 2025 were collected, and a total of 441 patients were included. In this study, elderly patients were defined as those aged 60–80 years. Osteoporosis was defined as QCT-measured lumbar vertebral trabecular bone mineral density <80 mg/cm³. According to QCT-measured bone mineral density, patients were divided into group A (147 cases, osteoporosis, bone mineral density <80 mg/cm³), group B (162 cases, low bone mass, 80 mg/cm³ ≤ bone mineral density ≤120 mg/cm³), and group C (132 cases, normal bone mass, bone mineral density >120 mg/cm³), and the prevalence of LDH among the three groups was compared. Based on patients’ past medical history, clinical manifestations, and imaging findings, group A was further divided into the LDH group (40 cases) and the non-LDH group (107 cases). Differences in general characteristics, laboratory indicators, bone mineral density parameters, lumbar–abdominal muscle endurance, and nutritional indicators between the two groups were compared. Multivariate Logistic regression analysis was used to identify independent risk factors for LDH in elderly patients with osteoporosis, and receiver operating characteristic (ROC) curves were plotted to evaluate the predictive performance of related indicators alone and in combination for LDH in elderly patients with osteoporosis. All statistical analyses were performed using GraphPad Prism 9.0 (GraphPad Software, USA).

**Results:**

The incidence of LDH in group A was significantly higher than that in groups B and C, and the differences among groups were statistically significant (P<0.05). Compared with the non-LDH group, the proportions of patients with bone mineral density <60 mg/cm³, estradiol (E2) level <18.35 pmol/L, and lumbar–abdominal muscle endurance <25 s were significantly higher in the LDH group (P<0.05). Multivariate Logistic regression analysis showed that bone mineral density <60 mg/cm³, E2 level <18.35 pmol/L, and lumbar–abdominal muscle endurance <25 s were independent risk factors for LDH in elderly patients with osteoporosis (P<0.05). ROC curve analysis showed that the areas under the curve (AUCs) for bone mineral density, E2 level, and lumbar–abdominal muscle endurance in predicting LDH in elderly patients with osteoporosis were 0.843, 0.847, and 0.826, respectively, while the AUC of the combined prediction was 0.892, and its predictive performance was significantly better than that of any single indicator (P<0.05).

**Conclusion:**

The incidence of LDH is significantly increased in elderly patients with osteoporosis, and decreased bone mineral density, reduced E2 levels, and impaired lumbar–abdominal muscle function are closely associated with the occurrence of LDH. Combined assessment of multiple indicators can significantly improve the predictive performance for the risk of LDH in elderly patients with osteoporosis, which is of great significance for early identification of high-risk populations and implementation of targeted interventions in clinical practice.

## 1 Introduction

With the continuous deepening of population aging, the disease burden of chronic degenerative disorders of the skeletal system in the elderly population has been increasing. Among them, osteoporosis, as one of the most common metabolic bone diseases in old age, has extended its adverse effects from a simple increase in fracture risk to multiple aspects such as decreased spinal structural stability, chronic pain, and limited motor function (1, 2). An increasing number of clinical observations (3, 4) have found that some elderly patients with osteoporosis often experience varying degrees of low back discomfort and decreased mobility even in the absence of obvious fractures, suggesting that osteoporosis may play an important role in the development of degenerative spinal diseases. LDH is one of the main causes of low back and leg pain in the elderly, and its occurrence is usually closely related to intervertebral disc degeneration, changes in vertebral load-bearing capacity, and imbalance of the overall spinal biomechanical environment. With increasing age, the water content of intervertebral discs decreases and elasticity weakens, making them more prone to structural changes under long-term loading (5, 6). Once LDH occurs in elderly individuals, symptoms are often prolonged and recovery is slow; in severe cases, walking ability and even activities of daily living may be affected, imposing a heavy burden on individuals, families, and healthcare systems (7).

In recent years, the potential association between osteoporosis and LDH has attracted increasing attention. On the one hand, some studies (8) have suggested that reduced bone mass may weaken the supportive effect of vertebral bodies on intervertebral discs, making discs more prone to abnormal deformation under load and thereby promoting degeneration and herniation; on the other hand, other studies (9) have argued that intervertebral disc degeneration is mainly related to changes in soft tissue structures and has limited association with bone mineral density. At present, conclusions from relevant studies remain inconsistent, which may be closely related to differences in study populations, insufficient sample sizes, and non-uniform methods for bone mineral density assessment. Regarding bone mineral density evaluation, although dual-energy X-ray absorptiometry (DXA) is widely used in clinical practice, its measurements in elderly populations with obvious spinal degenerative changes are easily affected by factors such as osteophyte formation and vertebral deformation, potentially leading to underestimation or overestimation of true bone mass levels (10). In contrast, QCT allows three-dimensional quantitative analysis of vertebral trabecular bone and can more accurately reflect true vertebral bone mineral density, showing certain advantages in studies related to the aging spine. However, studies that systematically analyze the occurrence of LDH in elderly populations based on QCT-derived bone mineral density stratification are still limited, and the available evidence remains insufficient. In addition, elderly patients with osteoporosis often present with multiple systemic changes, which may jointly influence the process of spinal degeneration. Nevertheless, most existing studies have focused on single factors, and there is a lack of comprehensive multifactorial analyses specifically targeting elderly patients with osteoporosis, particularly in terms of identifying high-risk populations and conducting risk assessment.

Based on the above background, this study retrospectively analyzed the clinical data of elderly patients who underwent lumbar QCT examination. Patients were grouped according to bone mineral density levels, and the occurrence of LDH under different bone density conditions was compared. This study further explored the influencing factors associated with LDH in elderly patients with osteoporosis, aiming to provide evidence for early identification of high-risk populations and optimization of prevention and treatment strategies in clinical practice.

## 2 Materials and methods

### 2.1 Study design and participants

**Figure 1:**
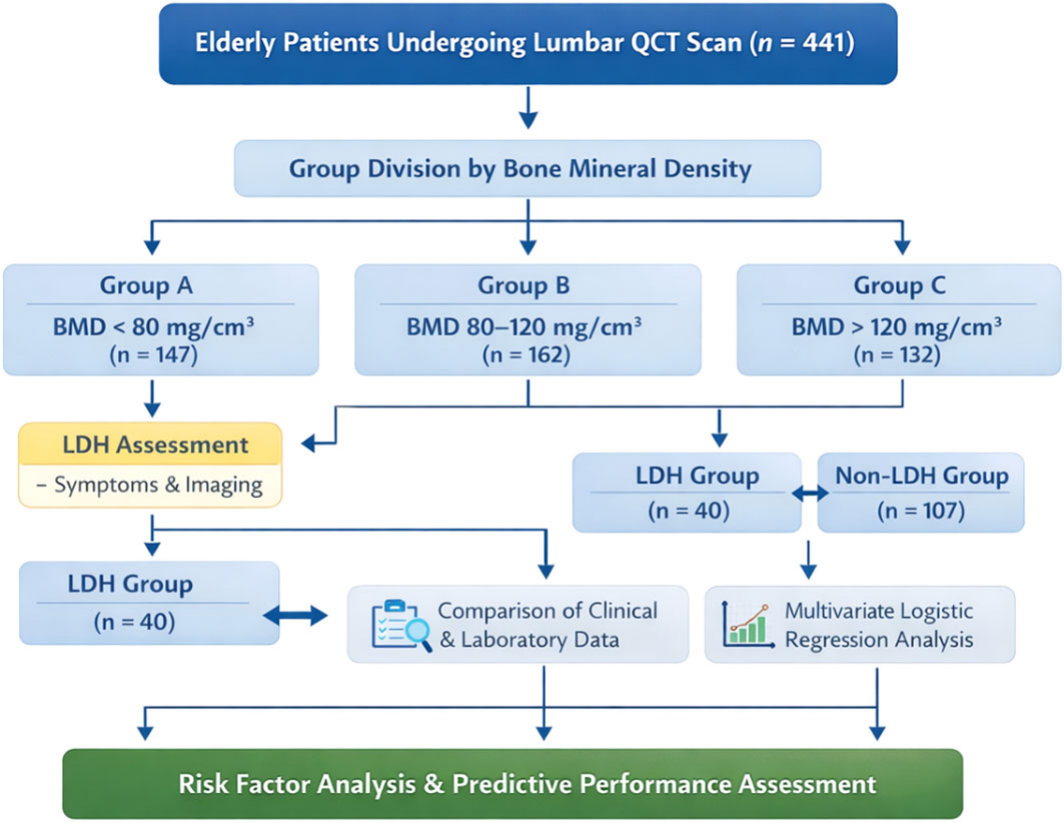
Flowchart of participant enrollment and study design.

A single-center retrospective case–control study design was adopted. Clinical data of 441 patients aged 60–80 years who underwent lumbar QCT examination at Tianshan Hospital of Traditional Chinese Medicine, Changning District, Shanghai, China, from January 2023 to January 2025 were consecutively collected. In this study, elderly patients were defined as those aged 60–80 years. Osteoporosis was defined according to the Chinese Guidelines for the Diagnosis and Treatment of Senile Osteoporosis (2018) (11) as lumbar QCT-measured vertebral trabecular bone mineral density <80 mg/cm³. Based on QCT-measured bone mineral density, patients were divided into group A (147 cases, osteoporosis, bone mineral density <80 mg/cm³), group B (162 cases, low bone mass, 80 mg/cm³ ≤ bone mineral density ≤120 mg/cm³), and group C (132 cases, normal bone mass, bone mineral density >120 mg/cm³). There were no statistically significant differences in sex, age, or body mass index (BMI) among the three groups (P>0.05), as shown in **Table 1**. Based on patients’ past medical history, clinical manifestations, and imaging findings, group A was further divided into the LDH group (40 cases) and the non-LDH group (107 cases) for analysis of influencing factors associated with LDH in elderly patients with osteoporosis. The flowchart of participant enrollment and study design is shown in **Figure 1** below.

**Table 1:** Comparison of baseline characteristics among the three groups.

Characteristic	Group A (n=147)	Group B (n=162)	Group C (n=132)	*F/x²*	*P*
Sex	–	–	–	0.364	0.847
Male	64 (43.54)	69 (42.59)	58 (43.94)	–	–
Female	83 (56.46)	93 (57.41)	74 (56.06)	–	–
Age (years)	72.84 ± 6.71	72.12 ± 6.98	71.69 ± 6.53	1.041	0.353
BMI (kg/m²)	23.62 ± 2.84	23.95 ± 3.06	24.08 ± 2.77	0.950	0.387

Note: Group A, osteoporosis; group B, low bone mass; group C, normal bone mass, classified according to QCT-measured lumbar vertebral trabecular bone mineral density.

### 2.2 Study population and eligibility criteria

Inclusion criteria were as follows: (1) patients aged 60–80 years, meeting the elderly age definition used in this study, regardless of sex; (2) patients who underwent lumbar QCT examination at the study hospital between January 2023 and January 2025; (3) patients with complete clinical data, including demographic information, medical history, laboratory test results, and imaging data; and (4) patients whose anonymized clinical data were available for retrospective analysis under the approval of the ethics committee.

Exclusion criteria were as follows: (1) previous lumbar or other spine surgery; (2) history of lumbar or spinal trauma; (3) vertebral compression fracture, previous vertebral fracture, spinal tumor, spinal infection, spinal tuberculosis, congenital spinal deformity, or other structural spinal diseases that could affect lumbar QCT measurement or LDH assessment; (4) concomitant diseases affecting bone metabolism, such as rheumatoid arthritis or diabetes mellitus; (5) severe cardiac, hepatic, or renal insufficiency, malignant tumors, or other serious systemic diseases; (6) long-term use of drugs affecting bone metabolism, such as glucocorticoids or parathyroid hormone; and (7) incomplete clinical, laboratory, or imaging data.

### 2.3 Ethical approval and data protection

This study was a single-center retrospective observational study, and the study design and reporting of results complied with the requirements of the Strengthening the Reporting of Observational Studies in Epidemiology (STROBE) guidelines. The study protocol was reviewed and approved by the Medical Ethics Committee of Tianshan Hospital of Traditional Chinese Medicine (ethical approval number: GKJC2407-HGLC). As this study was based on previously collected clinical data and did not involve additional interventions or increased risks to participants, written informed consent was waived with the approval of the ethics committee. The study strictly adhered to the Declaration of Helsinki (2013 revision) and relevant ethical regulations. All study data were obtained from the hospital electronic medical record system. During data collection and analysis, personal identifying information of patients was de-identified and access was restricted to authorized researchers only. Study results were presented in an aggregated manner and did not involve any identifiable individual information, thereby ensuring participant privacy and data security.

### 2.4 Methods

#### 2.4.1 Diagnostic criteria for LDH

The diagnosis of LDH was determined based on a comprehensive assessment of patients’ clinical symptoms and imaging findings. Imaging diagnosis was based on lumbar computed tomography (CT) or magnetic resonance imaging (MRI), showing posterior or posterolateral protrusion of the intervertebral disc with compression of the dural sac or nerve roots. Clinical manifestations such as low back and leg pain, radiating pain of the lower limbs, sensory abnormalities, or restricted movement were also considered. Two radiologists and clinicians with intermediate or higher professional titles independently evaluated the findings, and discrepancies were resolved through discussion to reach a consensus.

#### 2.4.2 Collection of general information and medical history

Before data extraction and statistical modeling, potential influencing factors for LDH in elderly patients with osteoporosis were prespecified according to clinical relevance and previous literature. These factors included demographic and baseline characteristics, namely sex, age, BMI, educational level, household income, and occupational type; lifestyle-related factors, including smoking history and alcohol consumption; laboratory indicators, including 25(OH)D_3_, serum calcium, serum phosphorus, and E2 levels; skeletal status, represented by QCT-measured lumbar bone mineral density; lumbar–abdominal muscle endurance; and nutritional status assessed using the NRS-2002 scale. Fall history within the previous year without acute spinal trauma or vertebral fracture, as well as regular anti-osteoporosis medication use before QCT examination, was also extracted from the medical records when available. Diabetes mellitus, vertebral fracture, and long-term systemic glucocorticoid or parathyroid hormone use were handled as exclusion criteria rather than influencing factors. Pain scores, ODI, and formal sarcopenia diagnosis were not included as prespecified influencing factors because they were not consistently documented in the retrospective medical records. These prespecified variables were used for baseline comparison between the LDH and non-LDH groups and for subsequent risk-factor analysis.

Demographic data and relevant medical history were systematically collected by reviewing electronic medical records in combination with standardized general information forms. The collected information included sex, age, BMI, educational level, household income, previous occupational type, smoking history, alcohol consumption, fall history, and anti-osteoporosis medication use. Smoking history was defined as smoking at least one cigarette per day for more than 3 months. Alcohol consumption history was defined as a daily alcohol intake >50 g or a weekly intake >350 g for no less than 6 months. Laboratory indicators, lumbar bone mineral density, lumbar–abdominal muscle endurance, and nutritional status were collected and assessed according to the procedures described below.

#### 2.4.3 Measurement of lumbar bone mineral density

Lumbar bone mineral density was assessed using QCT. Patients were placed in the supine position, and the scanning range covered the L1–L3 vertebral segments. After image acquisition using standardized scanning parameters, quantitative analysis of trabecular bone regions was performed using dedicated QCT analysis software (XTE6603C; Beijing Xiantong International Medical Technology Co., Ltd.). Cortical bone, osteophytes, and areas with obvious degenerative changes were avoided to reduce interference from degenerative alterations. Bone mineral density of the L1–L3 vertebral bodies was measured separately for each patient, and the average value was used as the final analytical indicator, expressed in mg/cm³.

#### 2.4.4 Laboratory indicators

Fasting venous blood samples were collected from all participants in the early morning. The level of 25-hydroxyvitamin D_3_ [25(OH)D_3_] was measured using fluorescence immunochromatography with a fluorescence immunoassay analyzer (P-4; WMT Medical Instruments (Hangzhou) Co., Ltd.). Serum calcium and phosphorus levels were determined using a spectrophotometer (AA-7003M; Beijing Dongxi Analytical Instrument Co., Ltd.), with methyl thymol blue colorimetry for calcium and the reduced molybdenum blue method for phosphorus. E2 levels were measured using magnetic particle chemiluminescence with a chemiluminescence analyzer (HomoG 100; Guangzhou Jinde Biotechnology Co., Ltd.). All procedures were performed strictly in accordance with the manufacturers’ instructions.

#### 2.4.5 Assessment of lumbar–abdominal muscle endurance

Lumbar–abdominal muscle endurance was assessed using a standard posture test (12). During the test, patients lay in the supine position, with both lower limbs extended and raised to approximately 30°, and the maximum time the position could be maintained was recorded in seconds (s) under standardized conditions. According to the duration, lumbar–abdominal muscle endurance was graded as follows: endurance ≥25 s was considered normal, and endurance <25 s was considered reduced.

#### 2.4.6 Nutritional status assessment

Nutritional status was evaluated using the Nutritional Risk Screening 2002 (NRS-2002) scale (13). The scale includes assessments of impaired nutritional status and disease severity, each scored from 0 to 3 points. An additional 1 point is added for patients aged ≥70 years, with a total score ranging from 0 to 7. A total score ≥3 indicates the presence of nutritional risk. All assessments were conducted by uniformly trained investigators to ensure consistency and reliability.

### 2.5 Statistical analysis

Data analysis was performed using GraphPad Prism 9.0 (GraphPad Software, USA). Before statistical analysis, all data were checked for completeness and consistency. Patients with missing key clinical, laboratory, or imaging data required for grouping, LDH diagnosis, or primary risk-factor analysis were excluded according to the eligibility criteria. Therefore, a complete-case analysis was performed, and no statistical imputation was applied.

Continuous variables were first tested for normality using the Shapiro–Wilk test. Variables with a normal distribution were expressed as mean ± standard deviation, and intergroup comparisons were performed using independent-samples t tests or one-way analysis of variance. Variables with a non-normal distribution were expressed as median (interquartile range), and intergroup comparisons were conducted using nonparametric tests. For descriptive baseline comparisons, continuous variables were retained as continuous data whenever appropriate. When clinically meaningful thresholds or study-defined cutoffs were required for risk-factor analysis, selected continuous variables were categorized before univariate and multivariate analyses, including bone mineral density, E2 level, 25(OH)D_3_ level, serum calcium level, serum phosphorus level, lumbar–abdominal muscle endurance, and NRS-2002 score. The cutoff values and category definitions were reported in the corresponding tables.

Categorical data were expressed as counts and percentages, and intergroup comparisons were performed using the χ² test. All prespecified influencing factors were first compared between the LDH and non-LDH groups among patients with osteoporosis. The presence of LDH was used as the dependent variable, and variables with statistical significance in univariate analysis were included in multivariate Logistic regression analysis to identify independent risk factors for LDH in elderly patients with osteoporosis. ROC curves were plotted, and AUCs were calculated to evaluate the predictive performance of related indicators alone and in combination for LDH in elderly patients with osteoporosis. A P value <0.05 was considered statistically significant.

## 3 Results

### 3.1 Comparison of LDH prevalence among the three groups

**Figure 2:**
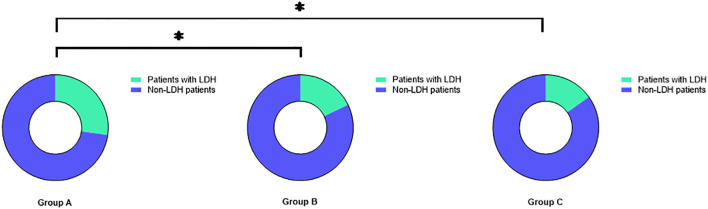
Comparison of LDH prevalence among the three groups. Note: Intergroup comparison, *P<0.05.

The prevalence of LDH in group A was 27.21% (40/147), which was significantly higher than that in group B (17.90%, 29/162) and group C (15.15%, 20/132), with statistically significant differences (P<0.05). However, there was no statistically significant difference in LDH prevalence between groups B and C (P>0.05), as shown in **Figure 2**.

### 3.2 Univariate analysis of factors associated with LDH in elderly patients with osteoporosis

Baseline characteristics and prespecified influencing factors among patients with osteoporosis are shown in **Table 2**. There were no statistically significant differences between the LDH and non-LDH groups in sex, age, BMI, educational level, household income, occupational type, smoking history, alcohol consumption, fall history within the previous year, 25(OH)D_3_ level, serum calcium level, serum phosphorus level, NRS-2002-defined nutritional risk, or anti-osteoporosis medication use (P>0.05). Compared with the non-LDH group, the proportions of patients with bone mineral density <60 mg/cm³, E2 level <18.35 pmol/L, and lumbar–abdominal muscle endurance <25 s were significantly higher in the LDH group (P<0.05).

**Table 2:** Baseline characteristics and prespecified influencing factors according to LDH status in elderly patients with osteoporosis.

Characteristic	LDH group (n=40)	Non-LDH group (n=107)	x²	P
Sex			0.933	0.333
Male	20 (50.00)	44 (41.12)		
Female	20 (50.00)	63 (58.88)		
Age (years)	73.46 ± 6.54	72.61 ± 6.78	0.681	0.497
BMI (kg/m²)	23.48 ± 2.79	23.67 ± 2.86	0.360	0.720
Educational level			1.490	0.222
High school and below	29 (72.50)	66 (61.68)		
Junior college and above	11 (27.50)	41 (38.32)		
Household income (CNY/month)			1.695	0.192
<10,000	25 (62.50)	54 (50.47)		
≥10,000	15 (37.50)	53 (49.53)		
Occupational type			2.461	0.116
Manual labor	23 (57.50)	46 (42.99)		
Non-manual labor	17 (42.50)	61 (57.01)		
Smoking history			0.587	0.443
Yes	15 (37.50)	33 (30.84)		
No	25 (62.50)	74 (69.16)		
Alcohol consumption			0.896	0.343
Yes	18 (45.00)	39 (36.45)		
No	22 (55.00)	68 (63.55)		
25(OH)D_3_ (ng/mL)			0.566	0.451
<20	27 (67.50)	65 (60.75)		
≥20	13 (32.50)	42 (39.25)		
Serum calcium (mmol/L)			0.412	0.520
<2.10	11 (27.50)	24 (22.43)		
≥2.10	29 (72.50)	83 (77.57)		
Serum phosphorus (mmol/L)			1.348	0.245
<0.90	16 (40.00)	32 (29.91)		
≥0.90	24 (60.00)	75 (70.09)		
NRS-2002 score (points)			1.112	0.291
<3	26 (65.00)	79 (73.83)		
≥3	14 (35.00)	28 (26.17)		
Bone mineral density (mg/cm³)			14.809	<0.001
<60	28 (70.00)	37 (34.58)		
≥60	12 (30.00)	70 (65.42)		
E2 (pmol/L)			11.439	<0.001
<18.35	25 (62.50)	34 (31.78)		
≥18.35	15 (37.50)	73 (68.22)		
Lumbar–abdominal muscle endurance (s)			10.000	0.001
<25	22 (55.00)	29 (27.10)		
≥25	18 (45.00)	78 (72.90)		
Fall history within the previous year			0.626	0.429
Yes	9 (22.50)	18 (16.82)		
No	31 (77.50)	89 (83.18)		
Anti-osteoporosis medication use			0.173	0.678
Yes	13 (32.50)	31 (28.97)		
No	27 (67.50)	76 (71.03)		

Values are presented as n (%) or mean ± standard deviation. LDH, lumbar disc herniation; BMI, body mass index; 25(OH)D_3_, 25-hydroxyvitamin D_3_; E2, estradiol; NRS-2002, Nutritional Risk Screening 2002.

### 3.3 Multivariate analysis of factors associated with LDH in elderly patients with osteoporosis

LDH status was used as the dependent variable (osteoporosis with LDH = 1, osteoporosis without LDH = 0). Variables with statistical significance in the univariate analysis shown in **Table 2** were included as independent variables and assigned values (**Table 3**) for multivariate Logistic regression analysis (**Table 4**). The results showed that bone mineral density <60 mg/cm³, E2 level <18.35 pmol/L, and lumbar–abdominal muscle endurance <25 s were independent risk factors for LDH in elderly patients with osteoporosis (P<0.05).

**Table 3:** Variable assignment.

Independent variable	Assignment
Bone mineral density	<60 mg/cm³ = 1; ≥60 mg/cm³ = 0
E2	<18.35 pmol/L = 1; ≥18.35 pmol/L = 0
Lumbar–abdominal muscle endurance	<25 s = 1; ≥25 s = 0

**Table 4:** Multivariate analysis of factors associated with LDH in elderly patients with osteoporosis.

Factor	*β*	*SE*	*Wald x²*	*P*	*OR*	*95%CI*
Bone mineral density <60 mg/cm³	1.425	0.396	13.281	<0.05	4.136	1.926–8.891
E2 <18.35 pmol/L	1.273	0.367	12.443	<0.05	3.562	1.774–7.159
Lumbar–abdominal muscle endurance <25 s	1.112	0.343	10.654	<0.05	3.034	1.553–5.928

### 3.4 Predictive performance of related factors alone and in combination for LDH in elderly patients with osteoporosis

**Figure 3:**
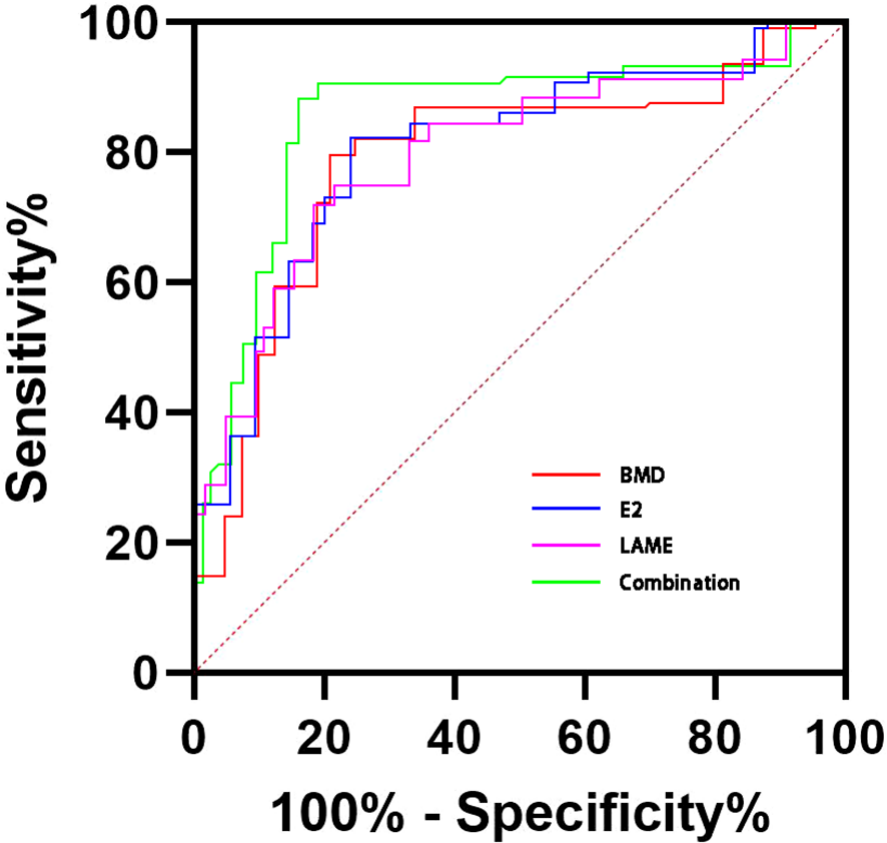
ROC curves of related factors alone and in combination for predicting LDH in elderly patients with osteoporosis. Note: BMD: Bone mineral density; LAME: Lumbar–abdominal muscle endurance.

ROC curve analysis showed that the AUCs of bone mineral density, E2 level, and lumbar–abdominal muscle endurance in predicting LDH in elderly patients with osteoporosis were 0.843, 0.847, and 0.826, respectively. The AUC of the combined prediction was 0.892, and its predictive performance was significantly better than that of any single indicator (P<0.05), as shown in **Table 5**; **Figure 3**.

**Table 5:** Predictive performance of related factors alone and in combination for LDH in elderly patients with osteoporosis.

Indicator	Cutoff value	AUC	95% CI	Sensitivity (%)	Specificity (%)	Youden index
Bone mineral density	63.15 mg/cm³	0.843	0.773–0.913	70.00	78.50	0.493
E2	18.80 pmol/L	0.847	0.781–0.914	62.50	83.20	0.462
Lumbar–abdominal muscle endurance	26.35 s	0.826	0.751–0.901	55.00	86.90	0.427
Combined assessment	—	0.892	0.835–0.948	77.50	85.00	0.635

## 4 Discussion

Based on lumbar QCT examination results, this study systematically analyzed the occurrence of LDH among elderly patients with different bone mineral density statuses and further explored the influencing factors and predictive performance for concomitant LDH in patients with osteoporosis. The results showed that, with the stepwise decrease in bone mineral density, the incidence of LDH exhibited a clear increasing trend, suggesting that osteoporosis may play an important role in the development of LDH in the elderly population. From a clinical perspective, these findings support the view that abnormalities in bone metabolism are not only key determinants of fracture risk but may also be deeply involved in the progression of degenerative spinal diseases, providing new insights for comprehensive risk assessment of spinal disorders in elderly patients with osteoporosis. In comparisons among different bone density groups, the incidence of LDH in group A was significantly higher than that in groups B and C, whereas the difference between groups B and C did not reach statistical significance. This finding suggests that the effect of decreased bone mineral density on the risk of LDH may exhibit a “threshold effect,” whereby the impact on spinal structural stability becomes more pronounced once bone loss progresses to the stage of osteoporosis. Previous studies have reported inconsistent conclusions regarding the relationship between bone mineral density and intervertebral disc degeneration, with some studies (14, 15) suggesting a limited association, while others (16, 17) supporting the notion that osteoporosis may promote disc degeneration. In the present study, QCT technology was used to quantitatively assess vertebral trabecular bone, effectively reducing the interference of degenerative osteophytes and endplate sclerosis on bone mineral density measurements. This approach rendered the comparison of LDH occurrence across different bone density states more objective and may partly explain discrepancies with some previous findings.

Further univariate and multivariate analyses conducted in patients with osteoporosis demonstrated that markedly reduced bone mineral density was an independent risk factor for concomitant LDH in elderly patients with osteoporosis. As bone mineral density decreases, destruction of the vertebral trabecular microarchitecture becomes more severe, with a reduction in trabecular number and increased disorganization, leading to a significant decline in the overall compressive and shear resistance of the vertebral bodies. Under the influence of daily activities, postural changes, and long-term axial loading, the supportive capacity of vertebral bodies for intervertebral discs is weakened, rendering the disc endplates more susceptible to microdamage. This, in turn, affects disc nutrition supply and metabolic balance. Such alterations in the mechanical and metabolic environment may provide favorable conditions for intervertebral disc degeneration and herniation, which is consistent with the theoretical concept of the “bone–disc unit” as an integrated degenerative structure (18–20). In addition to bone mineral density, this study also found that decreased E2 levels were closely associated with the risk of LDH in elderly patients with osteoporosis and emerged as an independent risk factor in multivariate analysis. E2 plays a crucial role in the regulation of bone metabolism, and its decline constitutes an important endocrine basis for the development of osteoporosis in the elderly, particularly in postmenopausal women (21). In recent years, an increasing number of studies (22, 23) have focused on the role of estrogen in intervertebral disc biology, suggesting that estrogen may not only indirectly influence spinal structure through effects on bone metabolism but may also directly participate in the regulation of disc cell metabolism. Some studies (24) have indicated that estrogen deficiency is associated with reduced anti-apoptotic capacity of disc cells, increased expression of inflammatory mediators, and decreased synthesis of extracellular matrix, thereby accelerating disc degeneration. The present study observed an association between reduced E2 levels and LDH occurrence at the population level, suggesting that endocrine factors may contribute to the development and progression of degenerative spinal disorders in elderly patients with osteoporosis through multiple pathways. Meanwhile, insufficient lumbar–abdominal muscle endurance was also identified as an independent risk factor for concomitant LDH in elderly patients with osteoporosis. As an essential component for maintaining dynamic spinal stability, the lumbar–abdominal muscle group plays a critical role in sharing disc loads and regulating spinal posture during daily activities (25, 26). In the elderly population, declines in physical activity, increased burden of chronic diseases, and changes in nutritional status contribute to generalized reductions in muscle mass and function, which are often more pronounced in patients with osteoporosis (27). When lumbar–abdominal muscle endurance is insufficient, the spine’s capacity to buffer external loads is diminished, resulting in increased mechanical stress on intervertebral discs and an accelerated process of degeneration and herniation (28). Previous studies examining the relationship between lumbar–abdominal muscle function and LDH (29, 30) have primarily focused on the general population or younger and middle-aged individuals. By incorporating this factor into the risk assessment framework for elderly patients with osteoporosis, the present study facilitates a more comprehensive understanding of LDH pathogenesis from a functional perspective.

It is noteworthy that, in univariate analysis, several sociodemographic characteristics, lifestyle factors, and certain laboratory indicators showed some differences between the LDH and non-LDH groups but did not reach statistical significance. These findings suggest that, among elderly patients with osteoporosis, the occurrence of LDH may be more strongly influenced by intrinsic factors such as bone metabolic status, endocrine changes, and spinal functional condition, whereas the effects of individual social or behavioral factors may be relatively limited. This also reflects the multifactorial and cumulative nature of degenerative spinal diseases in the elderly, which are driven by long-term interactions among multiple factors rather than by any single determinant. Regarding predictive performance, the results of this study indicated that bone mineral density, E2 levels, and lumbar–abdominal muscle endurance each demonstrated good discriminative ability in predicting concomitant LDH in elderly patients with osteoporosis, with further improvement observed when the three indicators were combined. This finding suggests that multidimensional integration of information can more comprehensively reflect the true risk status of spinal degeneration in elderly patients with osteoporosis and aligns with the current trend in chronic disease risk assessment shifting from single indicators to composite models (31, 32). The combined assessment approach shows strong clinical applicability and may provide valuable reference for early screening and stratified management of high-risk patients. From a clinical application perspective, these findings indicate that, in the comprehensive management of elderly patients with osteoporosis, clinicians should move beyond the traditional focus on bone mineral density or fracture presence alone and incorporate the risk of degenerative spinal diseases into the assessment framework. Comprehensive evaluation of bone metabolic status, endocrine function, and spinal functional condition may facilitate earlier identification of high-risk individuals and support the development of individualized prevention and treatment strategies. For example, alongside standardized anti-osteoporosis therapy, functional training aimed at improving spinal stability may have potential benefits in reducing the risk of LDH.

While affirming the findings of this study, several limitations should be objectively acknowledged. First, this was a single-center retrospective observational study, and all participants were derived from a single medical institution. Therefore, the sample composition may have been influenced by regional characteristics, population structure, and healthcare-seeking behaviors, and the applicability of the findings to other regions and populations requires further validation. Second, because the analyses were based on existing clinical records, some potentially relevant variables could not be fully captured due to limitations in medical record documentation. In addition, because patients with missing key data were excluded and no imputation was performed, potential selection bias related to complete-case analysis could not be fully avoided. Although BMI, smoking history, 25(OH)D_3_ level, fall history, and anti-osteoporosis medication use were extracted when available, other clinically important variables, including pain scores, ODI, formal sarcopenia diagnosis based on muscle mass or grip strength, and detailed information on anti-osteoporosis medication type, duration, and adherence, were not consistently documented in the retrospective records. Therefore, these variables could not be fully incorporated into the present analysis. Third, owing to the retrospective observational nature of the study, the results should be interpreted as associations rather than evidence of causality. Definitive causal relationships between the identified factors and LDH occurrence could not be established. Fourth, certain continuous variables were categorized in the statistical analysis. Although this approach enhanced clinical interpretability and practical applicability, it may have simplified the internal gradients of these variables and reduced sensitivity to subtle differences. Finally, although the combined assessment model demonstrated good discriminative ability, its stability and generalizability have not yet been externally validated in independent samples. Future multicenter prospective studies with larger sample sizes should include standardized assessment of pain severity, functional disability, sarcopenia, fall risk, and medication exposure, and should further validate and refine the predictive model.

## 5 Conclusion

The incidence of LDH in elderly patients with osteoporosis is significantly higher than that in populations with low bone mass or normal bone mass, indicating a close association between osteoporosis and the occurrence of LDH. Among patients with osteoporosis, markedly reduced bone mineral density, decreased E2 levels, and insufficient lumbar–abdominal muscle endurance were identified as independent risk factors for concomitant LDH, suggesting that abnormalities in bone metabolism, endocrine alterations, and spinal functional status may jointly contribute to the development of LDH in elderly patients with osteoporosis. Multivariate predictive analysis demonstrated that combined assessment of multiple indicators provides superior predictive performance compared with single indicators and enables a more comprehensive reflection of LDH risk in this population. In summary, risk assessment for LDH in elderly patients with osteoporosis should gradually shift from a sole focus on bone mineral density toward a multidimensional comprehensive evaluation encompassing bone metabolic status, endocrine function, and spinal functional condition. Early identification of high-risk individuals and implementation of targeted interventions may help reduce the incidence of LDH and improve spinal health and quality of life in elderly patients with osteoporosis.

## Data Availability

The original contributions presented in the study are included in the article/Supplementary Material, further inquiries can be directed to the corresponding author/s.
